# Supplementary material for: Psychophysiological correlates of science communicators
Source: PLoS One. 2025 Mar 26;20(3):e0320160. doi: 10.1371/journal.pone.0320160 (PMC11940683; doi:10.1371/journal.pone.0320160)
Supplement: S1 File — (DOCX) [file pone.0320160.s001.docx]

Supplementary Materials

**Psychophysiological correlates of science communicators.**

David Vagni^1*^, Gennaro Tartarisco^1*^, Simona Campisi^1^, Loredana Cerbara^2^, Marco Dedola^3^, Alessandra Pedranghelu^4^, Alexandra Castello^4^, Francesca Gorini^4^, Chiara Failla^1^, Marco Tullio Liuzza^5^, Antonio Tintori^2^, Giovanni Pioggia^1^, Marco Ferrazzoli^6,7§^, Antonio Cerasa^8,9§^

*3.1 Population characteristics*

For evaluating the level of self-esteem and prosociality scores in science communicators, we compared our score to two previous normative Italian samples. For self-esteem, two hundred controls are reported, *M* = 30.56, *SD* = 4.51 (Schmitt & Allik, 2005); in our sample, *M* = 35.06, SD = 4.05. The t-test led to, *t*(230) = 5.31, *p* < 0.001, MD = 4.50 ± 0.85. For prosociality, 2574 controls are reported, *M* = 3.52, *SD* = 0.64 (Caprara et al., 2005); in our sample, *M* = 3.93, SD = 0.50. The t-test led to, *t*(2604) = 3.59, *p* < 0.001, MD = 0.41 ± 0.11.

Therefore, we conclude that our participants were characterized by high levels of self-esteem and prosociality.

References

- Schmitt, D. P., & Allik, J. (2005). Simultaneous Administration of the Rosenberg Self-Esteem Scale in 53 Nations: Exploring the Universal and Culture-Specific Features of Global Self-Esteem. *Journal of Personality and Social Psychology, 89*(4), 623–642. [https://doi.org/10.1037/0022-3514.89.4.623](https://psycnet.apa.org/doi/10.1037/0022-3514.89.4.623)
- Caprara, G. V., Steca, P., Zelli, A., & Capanna, C. (2005). A new scale for measuring adults' prosocialness. *European Journal of Psychological Assessment*, *21*(2), 77-89. Doi: 10.1027/1015-5759.21.2.77

*3.5.3 Heart Rate Variability Analysis and Learning*

Here we reported additional analysis related to the relationship between HRV data and communication performance.

In the frequency domain, we found no multivariate or within-subject effects. Between-subject effect, using HF and LHFP as dependent variables, showed an effect of age, *F*(1,19) > 4.550, *p* < 0.046, *η*_p_^2^ > 0.193, and an effect of log(pub) while using LHFND, *F*(1,19) = 5.358, *p* = 0.032, *η*_p_^2^ = 0.220.

In the time domain, for the HR analysis, multivariate tests revealed a non-significant main effect of Phase and significant between-subjects effects for log(pub) and Gender, similar to the one found in previous analyses, and found also in the RR analysis.

*3.5.4 Heart Rate Variability Analysis and Engagement*

In the frequency domain, we found no multivariate or within-subject effects. Between-subject effects, using HF, LHFP, and LHFND as dependent variables, showed an effect of age, and an effect of log(pub) only while using LHFND, like previous analyses. For LHFND there was also a trend (*p* = 0.059) for the main effect of Phase.

In the time domain, we found no multivariate or within-subject effects, and significant between-subjects effects for log(pub) and Gender, like the one found in previous analyses.

In a subsequent analysis, no significant effects were observed using Agreement or Interest factors.

*3.6 Performance Prediction Based on HRV Indices*

A multivariate ANOVA was conducted with Gender as a factor, while age, log(pub), and the various HRV indices across different phases (denoted by subscripts) as covariates. The four performance-related variables (clarity, authority, learning, and engagement) served as the dependent variables. Box’s Test of Equality of Covariance Matrices and Levene’s Test of Equality of Error Variances were non-significant for all analyses, unless otherwise noted.

When LF and HF were introduced in covariation, multivariate analysis led to a significant effect of Age, *F*(4,12) = 6.201, *p* = 0.006, *η*_p_^2^= 0.674, HF_INT_, *F*(4,12) = 4.511, *p* = 0.019, *η*_p_^2^= 0.601, HF_POST_, *F*(4,12) = 7.592, *p* = 0.003, *η*_p_^2^= 0.717, LF_POST_, *F*(4,12) = 5.004, *p* = 0.013, *η*_p_^2^= 0.625. Levene’s Test of Equality of Error Variances was significant for Engagement, *p* < 0.001. The Corrected Model, led to a significant effect on Authority, *F*(9,15) = 4.209, *p* = 0.007, *η*_p_^2^= 0.716, Clarity, *F*(9,15) = 3.238, *p* = 0.022, *η*_p_^2^= 0.660, and Engagement, *F*(9,15) = 2.642, *p* = 0.046, *η*_p_^2^= 0.613. Between-subjects effect were significant for HF_INT_ on Clarity, *F*(1,15) = 4.827, *p* = 0.044, *η*_p_^2^= 0.243, HF_POST_ on Engagement, *F*(1,15) = 4.927, *p* = 0.042, *η*_p_^2^= 0.247, and marginally significant on Clarity, *F*(1,15) = 4.289, *p* = 0.056, *η*_p_^2^= 0.222. Furthermore, there was an effect of Age on Clarity, *F*(1,15) = 9.831, *p* = 0.007, *η*_p_^2^= 0.396, Authority, *F*(1,15) = 9.857, *p* = 0.007, *η*_p_^2^= 0.397, and Engagement, *F*(1,15) = 6.210, *p* = 0.025, *η*_p_^2^= 0.293.

Using RR as a covariate, the multivariate test was significant for age, *F*(4,15) = 4.492, *p* = 0.014, *η*_p_^2^= 0.545 and for RR_INT_, *F*(4,15) = 4.817, *p* = 0.011, *η*_p_^2^= 0.562. The Corrected Model, led to a significant effect on Authority, *F*(6,18) = 3.652, *p* = 0.015, *η*_p_^2^= 0.549, Clarity, *F*(6,18) = 3.740, *p* = 0.014, *η*_p_^2^= 0.555, and Engagement, *F*(6,18) = 4.632, *p* = 0.005, *η*_p_^2^= 0.607. Between-subjects effect were significant for RR_INT_ on Engagement, *F*(1,18) = 10.568, *p* = 0.004, *η*_p_^2^= 0.370, and marginally significant on Authority, *F*(1,18) = 3.876, *p* = 0.065, *η*_p_^2^= 0.177.

When LHFP, LHFND, HR, or SDNN were introduced in covariation, the only significant multivariate effect was for age.

*3.7 Participants Clustering based on HRV Indices*

Communicators were also divided into high stability (HS) and low stability (LS) groups using LF and HF in the three phases as features, leading to 19 HS and 6 LS participants. Cluster centers are presented in Table S4. No difference in any psychological or work-related indices was found between the two groups, but there was a significant difference in Clarity, *t*(23) = -2.783, *p* = 0.011, and Authority, *t*(23) = -2.721, *p* = 0.012. Comparing HRV and judgment clustering, we found that 100% of LS participants were in the LC group, *X^2^*(1, N = 25) = 11.842, *p* < 0.001, and 83% were also in the LA group, *X^2^*(1, N = 25) = 6.177, *p* = 0.023.

**Table S4. Heart Rate Variability Clusters**

| **Phase** | **Indices** | **Cluster** | | **Differences** | |
| --- | --- | --- | --- | --- | --- |
|  |  | **LS (N = 6)** | **HS (N = 19)** | **MD** | ***p*-value** |
| Pre-interview | LF | 1548 | 394 | 1154** | <0.001 |
|  | HF | 582 | 195 | 387** | 0.004 |
| Interview | LF | 545 | 429 | 116 | 0.588 |
|  | HF | 391 | 180 | 211 | 0.075 |
| Post-interview | LF | 2382 | 707 | 1674** | <0.001 |
|  | HF | 613 | 299 | 314** | 0.006 |

*K-Mean clustering* is *based on the six High Frequency (HF) and Low Frequency (LF) indices for the three phases. The two resulting clusters are named Low Stability (LS) and High Stability (HS), because the main distinguishing feature is the low difference between values in the LS group. MD = mean difference.*

The clustering of participants into high stability (HS) and low stability (LS) groups based on their LF and HF across phases revealed significant differences in clarity and authority. All LS participants were in the low clarity (LC) group, and a majority were in the low authority (LA) group. This strong association between HRV stability and performance judgments further supports that communicators who maintain stable autonomic responses are more likely to be perceived as clear and authoritative. This balanced state allows them to navigate the stress of public speaking with more stable autonomic responses, in contrast to those who experience greater autonomic instability across the different phases.
